# Long non-coding RNA LINC00470 in serum derived exosome: a critical regulator for proliferation and autophagy in glioma cells

**DOI:** 10.1186/s12935-021-01825-y

**Published:** 2021-03-04

**Authors:** Wenjia Ma, Yu Zhou, Min Liu, Qilin Qin, Yan Cui

**Affiliations:** Department of Neurosurgery, The Second Xiangya Hospital, Central South University, No. 139, Renmin Road, Changsha, 410011 Hunan People’s Republic of China

**Keywords:** LINC00470, Glioma, miR-580-3p, WEE1, PI3K/AKT/mTOR, Exosome, Autophagy, Proliferation

## Abstract

**Background:**

To explore the mechanism of LINC00470 in serum exosomes from glioma patients regulating the autophagy and proliferation of glioma cells.

**Methods:**

Exosomes were extracted from glioma patients (GBM-exo). Expression of LINC00470 in exosomes was analyzed with the clinicopathological characteristics of glioma patients. Glioma mouse model was established. The effects of LINC00470, miR-580-3p and WEE1 on cell autophagy and proliferation, as well as the activation of PI3K/AKT/mTOR pathway were measured. Dual luciferase reporter assay and RNA immunoprecipitation (RIP) were conducted to validate the binding of LINC00470 and miR-580-3p and of miR-580-3p and WEE1.

**Results:**

LINC00470 overexpressed in GBM-exo and associated with disease severity and postoperative survival time of glioma patients. GBM-exo deteriorated tumor progression in nude mice. Cells incubated with GBM-exo or transfected with pcDNA3.1-LINC00470/miR-580-3p inhibitor/pcDNA3.1-WEE1 had less autophagosome, downregulated LC3-II/LC3-I and Beclin1 expression levels and increased expression of p62 as well as strengthened proliferation ability. The PI3K/AKT/mTOR pathway was activated. LINC00470 competitively bound to miR-580-3p with WEE1.

**Conclusion:**

LINC00470 in GBM-exo can bind to miR-580-3p in glioma cells to regulate WEE1 expression and activate the PI3K/AKT/mTOR pathway, thereby inhibiting autophagy and enhancing the proliferation of glioma cells.

## Background

Glioma is the most frequently diagnosed primary brain tumor with the most terrible clinical prognosis among all brain tumors [1]. The advancement in diagnostic and surgical techniques improves the median survival and 2-year survival rate of glioma patients, however, the 5-year overall survival rate is only 9.8%, even with concomitant adjuvant temozolomide (TMZ) and radiotherapy [2–4]. Extracellular vesicles (EVs), including exosomes, have been reported to participate in cancer progression through facilitating the intercellular transfer of exosomal contents which are responsible for cell proliferation, migration, invasion, angiogenesis, and chemoresistance [5]. Fortunately, through analyzing the cargo loaded exosomes, the tumor growth could be tracked and predicted to allow early treatment for diseases [6]. Exosomes could carry a significant amount of nucleic acids, including mitochondrial DNA, mRNA, long noncoding RNAs (lncRNA), small nuclear RNAs and microRNA (miRNAs) [7]. Dysregulation of lncRNA is linked to cancer progression including apoptosis, metabolism, progression and metastasis [8].

As non-coding RNAs at the length of over 200 nucleotides, lncRNAs are viewed as peripheral biomarkers in cancers [9]. Recent study reported that many lncRNAs could regulate tumor progression through “lncRNA-miRNA-mRNA” mode [10]. As a dominant regulator of glioma cell autophagy, LINC00470 promoted the expression of ELFN2 through sponge of miR-101 to distract glioma cell autophagy [11]. Activation of autophagy might be a clinical objective that contributes to therapeutic efficiency of immunogenic chemotherapy and/or radiation therapy [12]. Previous studies have demonstrated that discoidin domain receptor tyrosine kinase 1 (DDR1) could sensitize glioma cells to therapies through its efficient induction of autophagic cell death [13, 14]. As an evolutionarily conserved process, autophagy is responsible for maintaining control of intracellular components through degradation mediated by lysosome, and in the processes of cellular and tissue repair, autophagy mainly protects against stresses and diverse pathologies including cancer [15]. Nevertheless, how LINC00470 regulates cell autophagy in glioma remains to be determined. Furthermore, cell autophagy in glioma involves the activation of PI3K/AKT/mTOR signaling network [16]. Inhibiting autophagy-related apoptosis by Cathepsin S has been proven as an effective therapeutic strategy for glioma through inhibition of PI3K/AKT/mTOR/p70S6K signaling pathway and activation of JNK signaling pathway [17]. But how PI3K/AKT/mTOR signaling pathway was activated and whether LINC00470 can regulate PI3K/AKT/mTOR signaling pathway in glioma are potential uncertainties for a better understanding of glioma.

In this study, we aimed to explore the role and mechanism of exosomal LINC00470 from serum of glioma patient in both cellular and animal models. The results demonstrated that LINC00470 in serum exosomes from glioma patients, through competing with WEE1 to bind miR-580-3p, could augment proliferation and impair the autophagy of glioma cells via activating PI3K/AKT/mTOR signaling pathway.

## Materials and methods

### Ethical statement

The study was conducted in accordance with the Declaration of Helsinki. The study protocol was approved by the Ethic Committee of the Second Xiangya Hospital, Central South University. All the patients provided their written informed consents. This design of clinical and animal experiments was approved by local commitment of the Second Xiangya Hospital, Central South University and was strictly implemented according to institutional guidelines. Experiments were performed in a humanistic way.

### Cell culture

Glioma cell lines (U251 and SWO-38) were obtained from the Cell Bank of Shanghai Institutes of Biochemistry and Cell Biology, Chinese Academy of Sciences. Cells were cultured in DMEM (Thermo Fisher Scientific, Wilmington, DE, USA) containing 10% FBS at 37 ℃ and 5% CO_2_ atmosphere.

### Exosome extraction and identification

We examined the expression of LINC00470 in the serum from 45 glioma patients and 10 health checkers (HCs) in our hospital. According to the WHO classification of tumors of central nervous system (CNS) (2016), the included glioma patients were classified into grade I ~ II (n = 18) and grade III ~ IV (n = 27). Exosomes derived from serum of glioma patients (patients in grade IV) and HCs were isolated by an ExoQuick kit (Invitrogen, Carlsbad, CA, USA) according to the manufacturer’s protocol, and named GBM-exo and HC-exo, respectively. Afterwards, the suspension was added onto the grid. After being placed for 1 min, the suspensions were negatively stained with 3% (w/v) sodium phosphotungstate solution for 5 min at room temperature and then washed and dried at room temperature for observation under a transmission electron microscope (TEM) (CM-120, Philips, Eindhoven, Netherlands).

Identification of exosomes: the expression levels of exosome markers (CD9, CD63 and CD81) were detected by Western blotting and flow cytometry (FCM) (primary antibodies for Western blotting were diluted at 1:1000). FCM: 100 μl of exosome suspension was incubated with primary antibodies against CD9 (ab2215, 1:200, Abcam), CD63 (ab59479, 1:200, Abcam) and CD81 (ab79559, 1:1000, Abcam) for 30 min and with corresponding secondary antibody (FITC-coupled goat anti mouse IgG, ab6785, 1:1000, Abcam) for 30 min before being detected by flow cytometry. Particle size distribution was analyzed by NanoSight NS300 instrument (Malvern, UK). After being labeled by PKH67 (Sigma-Aldrich, Merck KGaA, Darmstadt, Germany), the exosomes were incubated with DAPI (Sigma-Aldrich, Merck KGaA, Darmstadt, Germany) labeled U251 cells for 24 h to observe the uptake of exosomes by U251 cells. Exosomes were treated with RNase or RNase + TritonX-100 and then the expression of LINC00470 was measured.

### Animals

Specific pathogen free (SPF) BALB/c nude mice (n = 24, 4–6 weeks old, 16 ± 2 g) were purchased from Shanghai Laboratory Animal Science Center, Chinese Academy of Sciences. All materials including padding, water and pellet feed were sterilized by autoclave. All experimental mice were housed in a SPF laminar air flow room at constant temperature (22 ℃–26 ℃) and humidity (55 ± 5%).

### Mouse glioma model

U251 cells were infected with lentivirus containing luciferase and green fluorescent protein (GFP). FCM was used to select cell lines with stable expression of GFP-Luc. Nude mice were randomly separated into three groups (HC-exo group, GBM-exo group and sh-LINC00470-GBM-exo group) and then anesthetized through intraperitoneal injection of pentobarbital Sodium (60 mg/kg). After anesthesia, skin of the mouse heads was sterilized with iodine before the mice were fixed by stereotaxic apparatus. An incision was conducted longitudinally 3 mm right to the midline of the head, moving towards the animal’s ears to expose skull. A drill hole was made and a sterilized microinjector with U251-GFP-Luc cell suspension (1 × 10^6^) was fixed on the stereotaxic apparatus. The suspension was added through the stereotaxic apparatus after the syringe needle was inserted into a total depth of 2 mm below the surface of the brain. After injection, the drill holes were blocked with bone wax and the incisions were sutured before being sterilized. The mice were housed in their cage after anesthesia emergence. Mice in the GBM-exo group or HC-exo group were injected with 100 μl of U251-GFP-Luc cell suspension which had been cultured with serum exosome derived from glioma patients or HCs for 24 h, while mice in the sh-LINC00470-GBM-exo group were injected with U251-GFP-Luc cell suspension that had been incubated with serum exosomes derived from glioma patients for 24 h and transfected with sh-LINC00470.

### In vivo imaging system

The reactions of mice were regularly monitored and 10 μl/g of D-luciferin (at the concentration of 15 mg/ml) was intraperitoneally injected in mouse at the 0, 7th, 14th, 21th and 28th d of U251-GFP-Luc cell inoculation. In vivo imager (IVIS Spectrum, Caliper, USA) was used for color development and to observe changes in luminescence signal to analyze tumor progression.

### Hematoxylin eosin (H&E) staining and immunohistochemistry (IHC)

Tumor tissues were fixed with 4% of paraformaldehyde for 48 h before being sliced into paraffin sections (4 μm). The sections were subjected to H&E staining and IHC staining. After H&E staining, histopathological features of these sections were observed under an optical microscope. In brief, the sections were baked for 30 min and dewaxed with xylene before being washed with distilled water. Then, the sections were incubated with Ki-67 rabbit monoclonal antibody (ab16667, 1:200, Abcam, Cambridge, MA, USA) at 4 ℃ overnight after being washed with PBS for 1 min. The sections were cultured with corresponding secondary antibody for 1 h at room temperature after being washed with PBS three times. Following color development by DAB for 1 ~ 3 min, the nuclei was stained by hematoxylin for 1 ~ 3 min, and the sections were subjected to dehydration, permeabilization and mounting. Ki-67 is located in cell nucleus with yellow or brown granules within the cell nucleus. The percentage of Ki-67 positive cells was calculated in 5 high-power fields (× 400).

### Cell transfection

PcDNA3.1-LINC00470, pcDNA3.1-WEE1, sh-LINC00470, miR-580-3p mimic, miR-580-3p inhibitor and their negative controls (pcDNA3.1, mimic NC or inhibitor NC) were obtained from Shanghai GenePharma Co., Ltd (Shanghai, China). Transfection was performed by using Lipofectamine 2000 reagent (Invitrogen, Carlsbad, CA, USA) according to the manufacturer’s instruction. Cells were divided and named according to the transfected plasmids, including pcDNA3.1-LINC00470 group, pcDNA3.1-WEE1 group, pcDNA3.1 group, sh-LINC00470 group, sh-NC group, miR-580-3p mimic group, mimic NC group, miR-580-3p inhibitor group, and inhibitor NC group.

### qRT-PCR

TRIZOL (Invitrogen, Carlsbad, CA, USA) was used for RNA extraction and a reverse transcription kit (TaKaRa, Tokyo, Japan) for reverse transcription according to the instruction. The RNA expression levels of miR-580-3p, LINC00470 and WEE1 were detected by LightCycler 480 (Roche, Indianapolis, IN, USA) and the reaction conditions were prepared in accordance with the instruction of the fluorescent quantitative PCR kit (SYBR Green Mix, Roche Diagnostics, Indianapolis, IN). Parameters of thermal cycle were: 95 ℃ for 10 s, 45 cycles of 95 ℃ for 5 s, 60 ℃ for 10 s and 72 ℃ for 10 s, followed by extension at 72 ℃ for 5 min. Each quantitative PCR amplification was repeated for 3 times. U6 or GAPDH was taken as the internal reference. 2^−ΔΔCt^ method was used for statistical analysis. ΔΔCt = experiment group (Ct_target gene_–Ct_internal reference_)–control group (Ct_target gene_−Ct_internal reference_). The primers of the genes and its internal reference are quoted in Table 1.

**Table 1:** Primer sequences for quantitative reverse transcription polymerase chain reaction

Name of primer	Sequences
miR-580-3p-F	GACTATCGGGACATGTTA
miR-580-3p-R	GCAGGGTCCGAGGTATTC
U6-F	CTCTCGCTTCGGCAGCACA
U6-R	ACGCTTCACGAATTTGCGT
LINC00470-F	TTGGCAGCTGCTCTACAGTC
LINC00470-R	TGAAAATCCAGCCAGGGGTC
WEE1-F	TAGGTAAGGAGGCCTGTCCC
WEE1-R	TTTGAACACACGCAACGCAT
GAPDH-F	GCAAGGATGCTGGCGTAATG
GAPDH-R	TACGCGTAGGGGTTTGACAC

F, forward; R, reverse

### Western blotting

Total proteins were obtained using ratio-immunoprecipitation assay (RIPA) lysis buffer (Beyotime). After protein concentration was measured by a bicinchoninic acid (BCA) protein assay kit (Beyotime), loading buffer and protein were mixed and denatured in boiling water bath for 3 min. Extracted protein samples were separated by electrophoresis at 80 V for 30 min, and then the electrophoresis was run to 120 V for 1–2 h when bromophenol blue migrated to the separation gel. Membrane transferring was conducted in an ice bath at 300 mA for 60 min. Afterwards, the membrane was rinsed with washing solution for 1– 2 min followed by incubation with 5% skimmed milk at room temperature for 60 min or at 4℃ overnight. Primary antibodies against GAPDH (5174S, 1:1000), WEE1 (13084S, 1:1000), LC3-I/LC3-II (12741S, 1:1000), Beclin1 (3495S, 1:1000), p62 (88588S, 1:1000), PI3K (4249S, 1:1000), p-PI3K (p85, Tyr458) (17366S, 1:1000), AKT (4685S, 1:1000), p-AKT (Ser473) (4060S, 1:2000), mTOR (2983S, 1:1000), p-mTOR (Ser2448) (5536S, 1:1000) (Cell Signaling, Boston, USA) were incubated with the membranes at a shaking bed for 1 h at room temperature before being washed with washing solution for 3 × 10 min. Corresponding secondary antibody (goat anti-rabbit-IgG labeled with horseradish peroxidase, 1:5000, Beijing ComWin Biotech Co. Ltd, China, Beijing) was added for incubation for 1 h at room temperature followed by washing for 3 × 10 min. After color developement, the membranes were detected by chemiluminescence imaging system (Bio-rad).

### CCK-8

After cell transfection for 24 h, U251 cells in each group were inoculated onto 96 well plates and 100 μl of diluted suspension (1 × 10^6^/ml) was added into each well (three repetitions for each group). After incubation for 24 h, 48 h, 72 h and 96 h, 10 μl of CCK-8 reagent (Tokyo, Dojindo, Japan) was added into each well and incubated with the cells for 2 h. Then, the absorbance was detected at 450 nm wavelength.

### Clone formation assay

Twenty-four hours after cell transfection, cells were collected and digested using trypsin. The cells were centrifuged at 1500 rpm for 5 min at 25 ℃ and re-suspended with complete medium. Cells (500/well) were added onto 6 well plates containing 2 ml of pre-heated (37 ℃) complete medium and incubated at 37 ℃ with 5% CO_2_ for 2–3 weeks. Cells were ceased to culture after cell clones could be seen by naked eyes in the 6 well plates. Then, the culture medium was removed and the cells were washed with PBS twice carefully. Methanol (1.5 ml/well) was added to fix the cells for 15 min. After fixation, the methanol was discarded, and Giemsa stain (1 ml) was added along the well to stain cells for 20 min in dark. Cells were then washed in running water to remove the excessive dye. Turn the 6-well plate upside-down on absorbent paper. The number of clones was counted under a microscope (low power lens).

### Detection of cell cycle by FCM

U251 cells, after the transfections for 24 h, were subjected to centrifugation at 1000 rpm for 10 min before being washed with 3 × PBS. Cell suspension was fixed using ethyl alcohol (70%) for 1 h and then centrifuged to discard the ethyl alcohol. After being rinsed with PBS, the cells were incubated with DNAzyme-free RNAase (final concentration at 100 μg/ml) and propidium iodide (PI) (final concentration at 20 μg/ml) in dark for 30 min. DNA content was measured using FCM (EPICS-XL, Beckerman Coulter, USA).

### Monodansylcadaverin (MDC) staining

Transfected U251 cells were incubated in fresh culture medium containing MDC (50 μM) in a 37℃ incubator with 5% CO_2_ for 20 min in dark, followed by 2 × PBS washes. U251 cells were then stained by DAPI for 5 min away from light and washed in PBS twice. U251 cells were observed and photographed under an inverted microscope, and the fluorescence intensity was analyzed. Autophagy was activated in response to 12 h treatment of Rapamycin (200 nM) (Rapa, Sigma-Aldrich, Merck KGaA, Darmstadt, Germany) treatment.

### Dual luciferase report assay

The binding sites of LINC00470 and miR-580-3p were predicted by DIANA TOOLS LncBase Predicted v.2 (http://carolina.imis.athena-innovation.gr/diana_tools/web/index.php?r=lncbasev2/index-predicted) and the binding sites of miR-580-3p and WEE1 by starBase (http://starbase.sysu.edu.cn/). Wild type (wt-LINC00470 and wt-WEE1) and mutant type (mut-LINC00470 and mut-WEE1) of the binding sites were designed and synthesized. Luciferase report vectors (pGL3-Promoter) were inserted with the wild type or mutant type sequences, prior to co-transfection with miR-580-3p mimic (30 nM) or it negative controls (30 nM) into HEK239T cells (Shanghai Sixin Biotechnology Co. Ltd). Renilla luciferase activity was taken as the internal control. Ratio between firefly luciferase activity and renilla luciferase activity was the relative luciferase activity.

### RNA immunoprecipitation (RIP)

Preparation of cells: collected cells were washed with ice-cold PBS twice and centrifuged at 1500 rpm for 5 min before being lysed with RIP lysis buffer. Preparation of magnetic beads: the 50 μl of resuspended beads were pipetted into a centrifuge tube. RIP wash buffer (500 μl) was added into the tube and then the tube was fully shaken. The tube was placed on the magnetic rack and moved left-to-right at 15 degree for bead absorption before the cell suspension was removed. The aforementioned procedures were repeated once more to wash beads. The beads were re-suspended with 100 μl of RIP wash buffer and then incubated with 5 μg of Ago2 antibody (2897S, 1:50, Cell Signaling, Boston, USA) at room temperature for 30 min. Afterwards, the tube was placed on the magnetic rack to remove the supernatant. RIP wash buffer (500 μl) was added, and the tube was shaken by vortexing to discard the supernatant, which was repeated once more. RIP wash buffer (500 μl) was added, and the tube was shaken by vortexing and then placed on ice. The tube containing the magnetic beads was placed on the magnetic rack to remove the supernatant. After that, 900 μl of RIP Immunoprecipitation Buffer was added into the tube. Prepared cell lysate was unfroze and centrifuged at 14,000 rpm and 4 ℃ for 10 min. Cell suspension (100 μl) was added into the tube containing bead-antibody complexes to make the final volume l ml. The mixture was incubated at 4 ℃ overnight and shortly centrifuged. After that, the tube was placed on the magnetic rack and the supernatant was removed. RIP wash buffer (500 μl) was added into the tube on the magnetic rack, after which the supernatant was removed and the complex was washed 6 times. RNA purification: each immunoprecipitate was resuspended in 150 μl of Proteinase K Buffer bead-antibody complex. All samples were incubated at 55 ℃ for 30 min. After incubation for 30 min, the tubes were placed in the magnetic rack and the supernatant was removed. After extraction of RNA, the expression levels of miR-580-3p, LINC00470 and WEE1were measured by qRT-PCR.

### Statistical analysis

Statistical analysis was carried out using GraphPad 7.0. Measurement data are presented in the form of mean ± standard deviation. Comparison between two groups was analyzed using *T* test, while Dunnett’s multiple comparisons test was used for multiple comparisons after One-way analysis of variance. Survival analysis was conducted by Kaplan–Meier, and chi-square test or T test was used to analyze the relationship of LINC00470 with the clinicopathological characteristics of glioma patients. *P* < 0.05 was viewed as statistically significant.

## Results

### Identification of exosome in serum

**Fig. 1:**
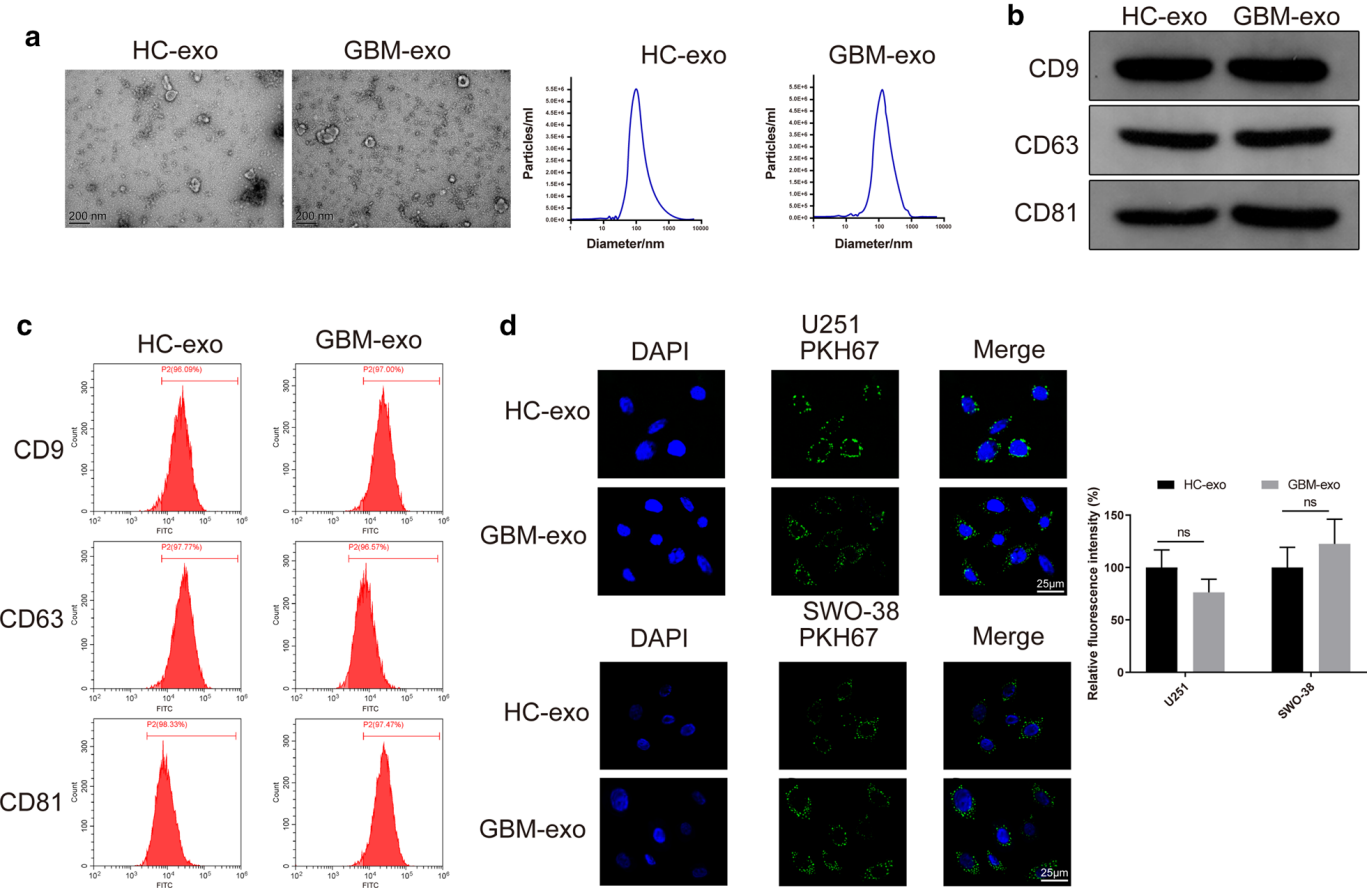
Identification of exosomes in serum of patients with glioma and HCs. Note: Serum exosomes of glioma patients and HCs were observed by TEM, and the particle size distribution was analyzed (**a**); Detection of the expression levels of exosome markers (CD9, CD63 and CD81) by Western blotting (**b**) and FCM (**c**). Uptake of PHK67 labeled exosomes in serum by U251 and SWO-38 cells by fluorescence microscope (**d**). GBM-exo, exosomes derived from patients with glioma; HC, health checker; TEM, transmission electron microscope; FCM, flow cytometry

Exosomes extracted from serum of glioma patients and HCs were observed under a TEM. Particle size analysis showed that the size distribution and concentration of exosomes from glioma patients and HCs had no significant differences (Fig. 1a). Western blotting and FCM revealed the positive expression levels of CD9, CD63 and CD81 in the extracted exosomes (Fig. 1b–c). To observe the uptake of exosome by U251 cells and SWO-38 cells, a fluorescence microscope was used to detect the location of PKH67 labeled exosome in cells. We noted that PKH67 labeled exosomes were transferred into the cytoplasm of U251 and SWO-38 cells after being incubated with U251 and SWO-38 cells, and the PKH67 fluorescence intensities of exosomes from glioma patients and HCs had no substantial differences (Fig. 1d). These results suggested that U251 and SWO-38 cells could ingest exosomes.

### LINC00470 expression in serum exosomes from glioma patients correlated with disease progression and survival

**Fig. 2:**
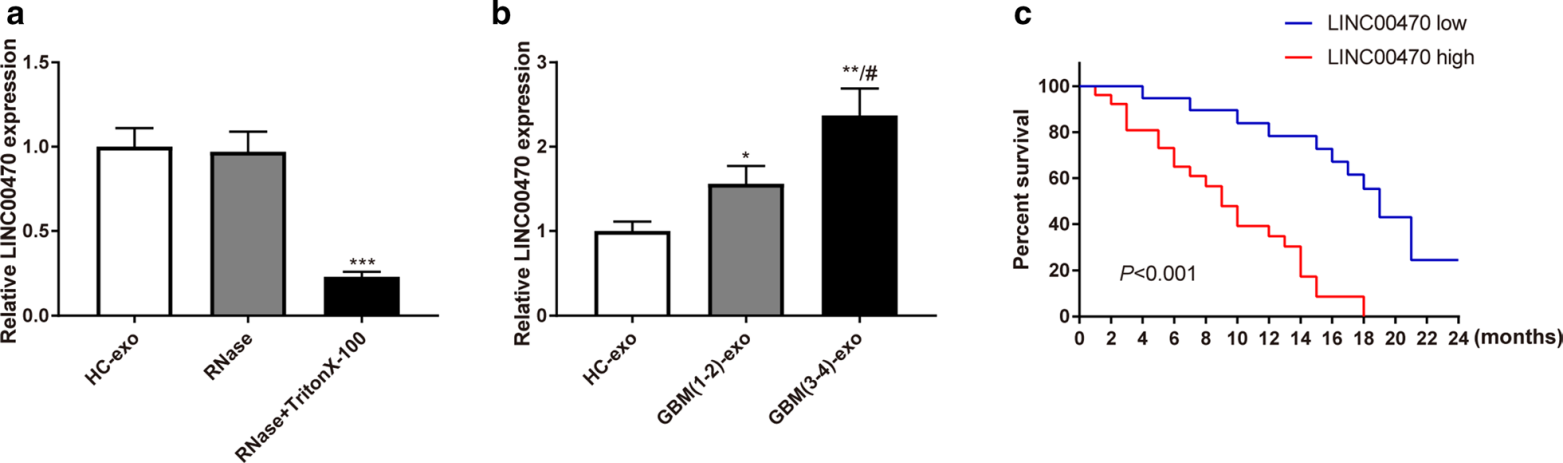
LINC00470 expression in serum exosomes was negatively correlated with survival of glioma patients. Note: Expression of LINC00470 in HCs’ serum exosomes treated by RNase or RNase + TritonX-100 was detected by qRT-PCR (**a**). Detection of LINC00470 expression in serum exosomes of glioma patients and HCs by qRT-PCR (**b**). Kaplan–Meier method was used to examine the correlation of LINC00470 expression in serum exosomes of glioma patients with survival time (**c**). ** P < 0.01, *** P < 0.001, compared to HC-exo group. #* P* < 0.05, compared to glioma (grade I ~ II) group. GBM-exo, exosomes derived from patients with glioma; HC, health checker

Previous studies have proved overexpression of LINC00470 in glioma [11, 18]. We extracted exosomes from the serum of glioma patients and measured the expression of LINC00470 in the exosomes to further analyze whether the expression of LINC00470 correlated with the clinicopathological characteristics of glioma patients. Firstly, to detect the expression of LINC00470 in serum exosomes of HCs, extracted exosomes were treated with RNase or RNase + TritonX-100. There was no detectable change in LINC00470 expression in RNase-treated serum exosomes, while the expression of LINC00470 in exosomes was substantially declined after RNase + TritonX-100 treatment, compared to the HC-exo group (Fig. 2a, P < 0.001). The results indicated that exosomes were encased with phospholipid membrane.

Next, LINC00470 expression in serum exosomes from HCs and glioma patients was detected and compared. We found that LINC00470 expression was the lowest in serum exosomes from HCs, and glioma patients in grade III–IV had the highest LINC00470 expression (Fig. 2b, P < 0.05).

Later, we analyzed the correlation of LINC00470 expression with the clinicopathological characteristics of glioma patients. The expression of LINC00470 in glioma patients were measured and recorded to calculate the average expression of LINC00470 in glioma patients. According to the average expression of LINC00470, glioma patients were classified into LINC00470 high expression group (n = 26) and LINC00470 low expression group (n = 19). We collected the clinicopathological characteristics of these patients, including gender, age at diagnosis, disease staging, KPS score of glioma patients, MGMT promoter methylation and IDH mutation. As listed in Table 2, chi-square test and T test demonstrated that LINC00470 expression didn’t associate with gender (*P* = 0.787), age at diagnosis (*P* = 0.207), MGMT promoter methylation (*P* = 0.3792) or IDH mutation (*P* = 0.2807) but significantly correlated with disease staging (*P* = 0.0003) and KPS score (*P* = 0.044). Patients in the LINC00470 low expression group had moderate disease development and higher KPS score than those in the LINC00470 high expression group. Moreover, we followed up the prognosis of these patients (n = 45) for 24 months. The median survival of patients in the LINC00470 high expression group was 9 months while that in the low expression group was 19 months. The analysis by Kaplan–Meier showed LINC00470 was negatively associated with the survival of glioma patients (Fig. 2c, P < 0.001). Aforementioned results suggested that LINC00470 was up-regulated in serum exosomes from glioma patients and correlated with disease progression and postoperative survival of glioma patients.

**Table 2:** Association of LINC00470 expression with clinicopathological characteristics of GBM patients

Pathological characteristics	LINC00470 low	LINC00470 high	*P* values
Gender (F/M)	8/11	12/14	0.787
Age (years)	46.37 ± 9.67	50.42 ± 11.34	0.207
Grade (1–2/3–4)	6/13	4/22	0.0003***
KPS score (≥ 70/ < 70)	9/10	5/21	0.044*
MGMT promoter methylation (±)	9/10	10/16	0.3792
IDH mutation (±)	6/13	4/22	0.2807

F, female; M, male; KPS score, karnofsky performance scale score; GBM, glioma; MGMT, O6-methyl guanine-DNA methyltransferases; IDH, isocitrate dehydrogenase; KPS < 70 points means relative severe disease progression and KPS ≥ 70 points refers to relative moderate disease progression

* *P* < 0.05, *** *P* < 0.001

### LINC00470 in serum exosomes implicated in tumor progression in primary glioma mouse models

**Fig. 3:**
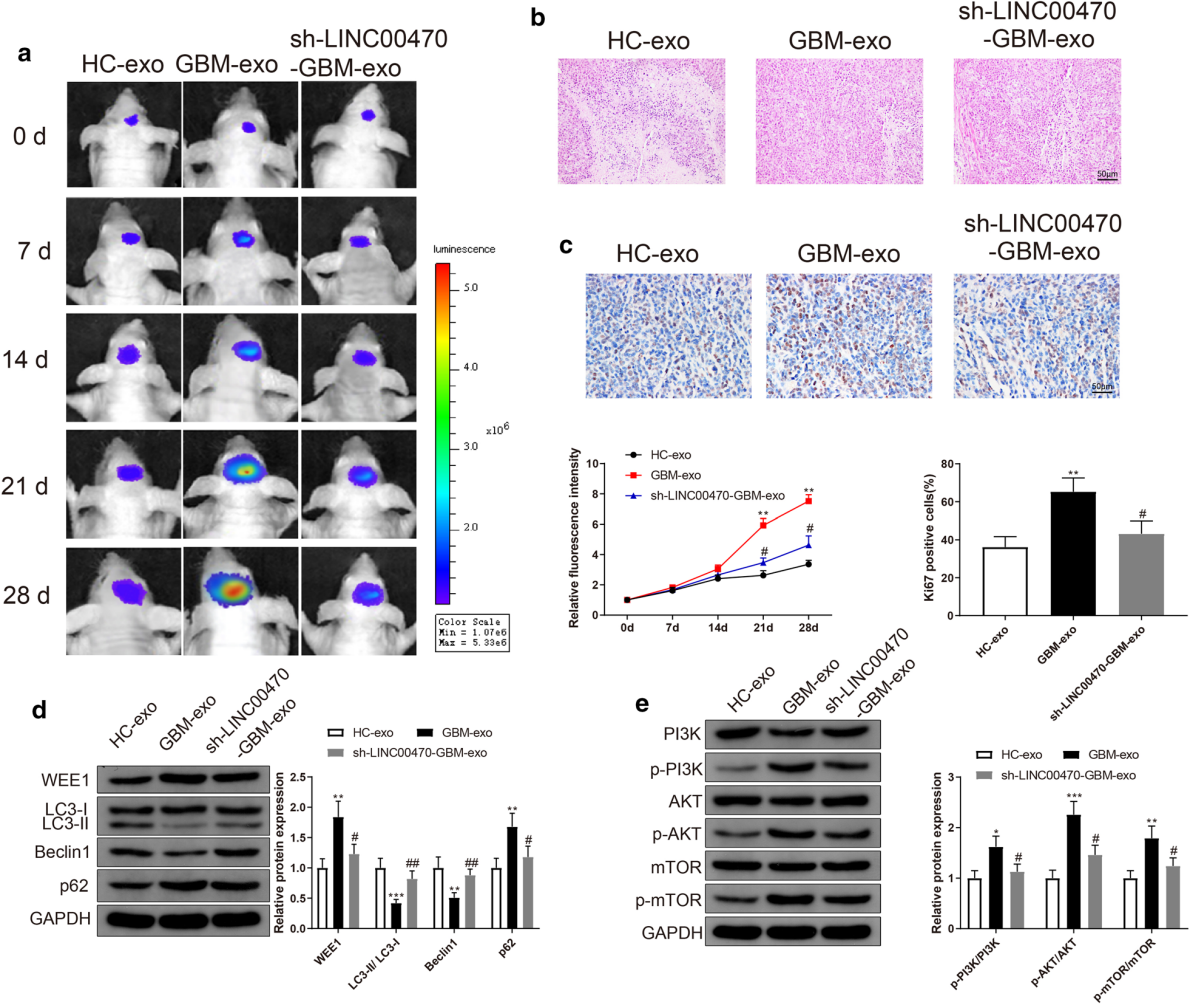
Exosomes from serum of glioma patients affected tumor formation in nude mouse. Note: In vivo imaging analyzed fluorescence intensity in brains of nude mice (**a**). H&E staining showing the morphology of mice injected with serum exosomes of glioma patients (**b**). IHC staining was performed to measure the expression of Ki67 (**c**). Western blotting was used to detect the expressions of autophagy related proteins (LC3-II/LC3-I, Beclin1 and p62) as well as WEE1 expression (**d**) and PI3K/AKT/mTOR pathway related proteins (**e**). * *P* < 0.05, ** *P* < 0.01, *** *P* < 0.001, compared to HC-exo group. GBM-exo, exosomes derived from patients with glioma; H&E, Hematoxylin eosin; IHC, immunohistochemistry

We subsequently identified the effect of exosomal LINC00470 in glioma patient serum on the growth of primary glioma in nude mice. Human glioma cells U251 were used for the establishment of glioma model [19]. U251-GFP-Luc cells or U251-GFP-Luc cells transfected with sh-LINC00470 were inoculated into nude mice after 24 h of incubation with serum exosomes from HCs or glioma patients. After 0, 7, 14 d of model establishment, in vivo imaging system showed that the fluorescence intensities among the HC-exo group, GBM-exo group and sh-LINC00470-GBM-exo group had no significant differences (Fig. 3a). After 21 d and 28 d of model establishment, the fluorescence intensity was remarkably increased in the GBM-exo group compared with the HC-exo group, while the fluorescence intensity in the sh-LINC00470-GBM-exo group was substantially decreased when compared to that in the GBM-exo group (Fig. 3a, P < 0.05).

H&E staining for the tumor tissues of nude mice revealed that cells in the GBM-exo group arranged more densely and had increased cell size when compared to the HC-exo group or sh-LINC00470-GBM-exo group (Fig. 3b). Consistently, IHC verified that the GBM-exo group had increased Ki67 positive cells compare with the HC-exo group or sh-LINC00470-GBM-exo group (Fig. 3c, P < 0.05). Collectively, these results indicated that exosomes isolated from serum of glioma patients enhanced the tumorigenesis of U251 cells in nude mice, while LINC00470 knockdown inhibited the growth of xenograft tumor.

Furthermore, we also found that LC3-II/LC3-I and Beclin1 expression levels were decreased and p62 expression was increased in the GBM-exo group compared with those in the HC-exo group or sh-LINC00470-GBM-exo group (Fig. 3d, P < 0.05), which suggested that exosomes derived from the serum of glioma patients can inhibit cell autophagy while LINC00470 knockdown could abolish GBM-exo induced autophagy inhibition. Moreover, protein WEE1 expression was enhanced in the GBM-exo group when compared with that in the HC-exo group or sh-LINC00470-GBM-exo group (Fig. 3d, P < 0.05). In addition, the expression levels of p-PI3K/PI3K, p-AKT/AKT and p-mTOR/mTOR in the GBM-exo group were much higher than those in the HC-exo group and sh-LINC00470-GBM-exo group (Fig. 3e, P < 0.05), indicating that serum exosomes from glioma patients could activate the PI3K/AKT/mTOR pathway and LINC00470 could partially offset the activation.

### Exosomal LINC00470 inhibits autophagy and promotes proliferation of glioma cells

**Fig. 4:**
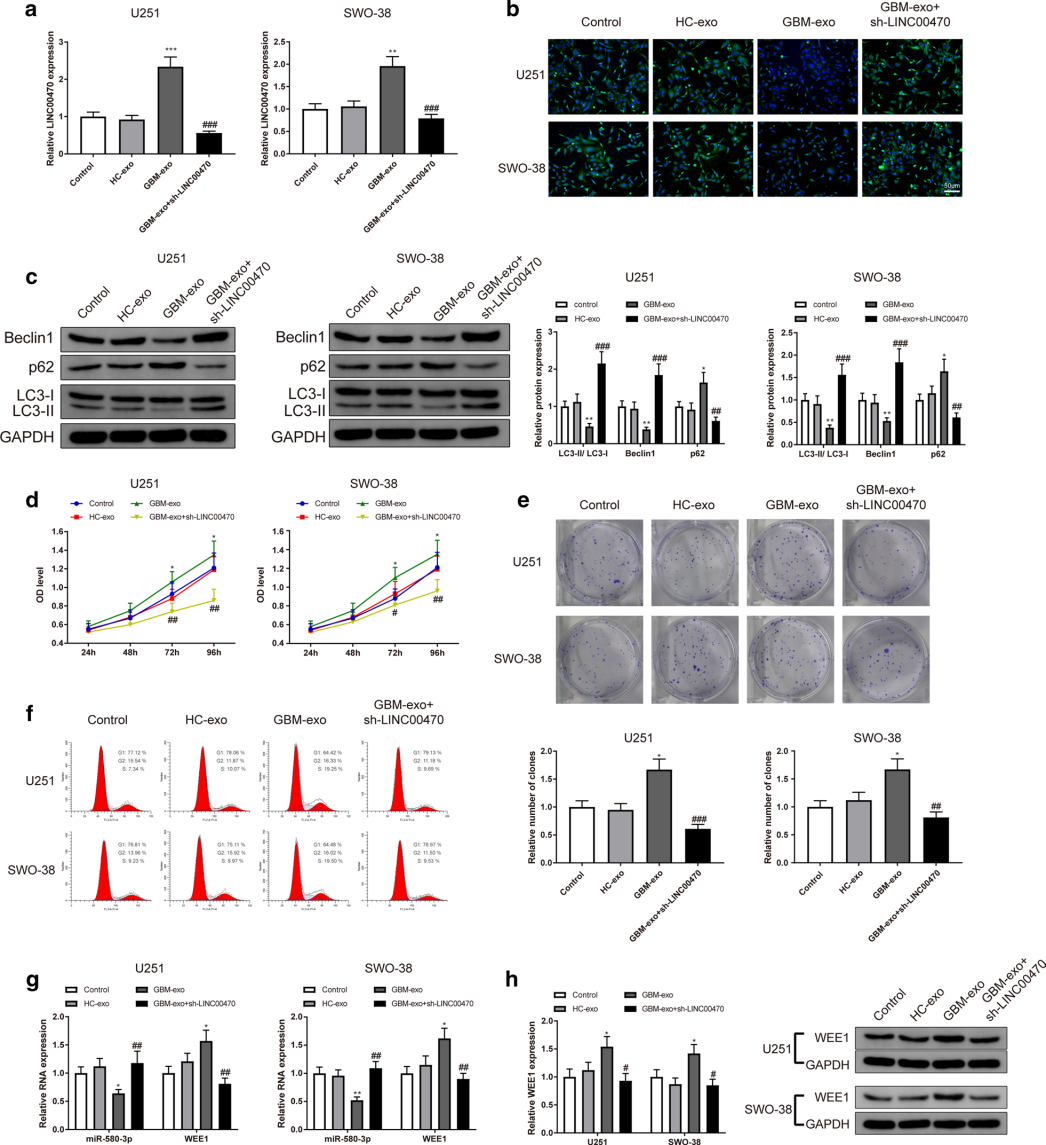
Exosomal LINC00470 in serum of glioma patients mediated the autophagy and proliferation of glioma cells. Note: U251 and SWO-38 cells were firstly incubated with GBM-exo or HC-exo and transfected with sh-LINC00470 before the regulatory role of LINC00470 in exosome on glioma cell was assessed. Expression of LINC00470 was detected by qRT-PCR (**a**). Number of acidic autophagosomes was shown through MDC staining (**b**). Western blotting analyzed autophagy-related proteins expression levels including LC3-II/LC3-I, Beclin1 and p62 (**c**). Cell proliferation was examined by CCK8 (**d**) and clone formation assay (**e**) respectively. Cell cycle was revealed by FCM (**f**). RNA and protein expression levels of miR-580-3p and WEE1 were detected by qRT-PCR (**g**) and Western blotting (**h**) respectively. * *P* < 0.05, ** *P* < 0.01, ***P < 0.001, compared to HC-exo group. # *P* < 0.05, ## *P* < 0.01, ###*P* < 0.001, compared to GBM-exo group. GBM-exo, exosomes derived from patients with glioma; HC, health checker; MDC, monodansylcadaverin staining; FCM, flow cytometry

The effects of exosomal LINC00470 on the autophagy and proliferation of U251 and SWO-38 cells were explored on cellular background. U251 and SWO-38 cells were incubated with HC-exo or GBM-exo before qRT-RCR was applied to measure the expression of LINC00470. qRT-RCR manifested that LINC00470 expression in the GBM-exo group was much higher than that in the HC-exo group (Fig. 4a, P < 0.05). Compared to the HC-exo group, the GBM-exo group showed decreased acidic autophagosomes (Fig. 4b), declined expression levels of LC3-II/LC3-I and Beclin1, increased expression of p62 (Fig. 4c, P < 0.05), strengthened proliferation ability (Fig. 4d–e, P < 0.05) and less cells in G1 phase (Fig. 4f, P < 0.05).

To ascertain the role of LINC00470 in exosomes derived from serum of glioma patients, sh-LINC00470 was transfected into U251 and SWO-38 cells after the cells were incubated with GBM-exo for 24 h. Compared to the GBM-exo group, the GBM-exo + sh-LINC00470 group had decreased LINC00470 expression (Fig. 4a, P < 0.05), increased acidic autophagosomes (Fig. 4b), upregulated expression levels of LC3-II/LC3-I and Beclin1, declined expression of p62 (Fig. 4c, P < 0.05), downregulated proliferation ability (Fig. 4d–e, P < 0.05) and increased number of G1-phase cells (Fig. 4f, P < 0.05). Aforementioned results supported that GBM-exo could inhibit the autophagy of U251 and SWO-38 cells and promote their proliferation, while downregulation of LINC00470 can reverse the effect of GBM-exo, indicating that it was LINC00470 in GBM-exo that mediated the autophagy and proliferation of U251 and SWO-38 cells.

Furthermore, miR-580-3p expression was decreased, while WEE1 expression was increased in the GBM-exo group compared with those in the HC-exo group (Fig. 4g–h, P < 0.05). However, the expression of miR-580-3p was increased and the expression of WEE1 was decreased in the GBM-exo + sh-LINC00470 group when compared with those in the GBM-exo group (Fig. 4g–h, P < 0.05).

### Exosomal LINC00470 promotes the proliferation of glioma cells through regulating autophagy

We previously confirmed that LINC00470 can suppress autophagy and enhance the proliferation of U251 and SWO-38 cells; however, whether LINC00470 could promote the proliferation of U251 and SWO-38 cells via inhibiting autophagy was still unclear. Therefore, we used Rapa to activate autophagy. U251 and SWO-38 cells were interfered with 200 nM of Rapa for 12 h to activate autophagy after the cells had been transfected with pcDNA3.1-LINC00470 for 24 h.

**Fig. 5:**
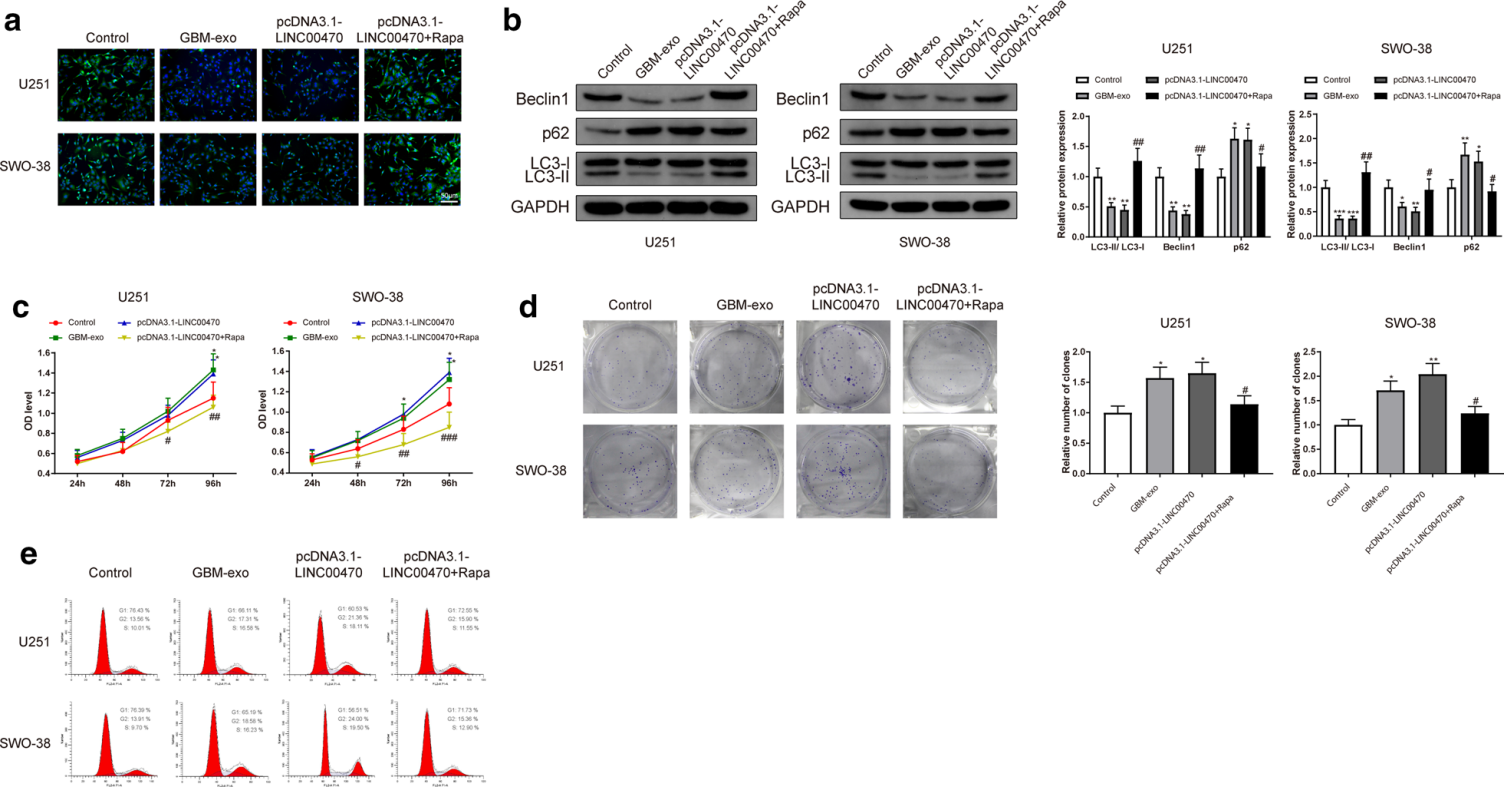
Exosomal LINC00470 promoted the proliferation of glioma cells through regulating autophagy. Note: U251 and SWO-38 cells were incubated with GBM-exo or transfected with pcDNA3.1-LINC00470 or treated with Rapa. MDC staining was used to detect acidic autophagosomes (**a**). Detection of the expression of autophagy-related proteins, LC3-I, Beclin1 and p62 was conducted by Western blotting (**b**). Proliferation was examined through CCK8 and clone formation assay (**c**–**d**). FCM examined the effect of LINC00470 and autophagy on cell cycle of glioma cells (**e**). * *P* < 0.05, ** *P* < 0.01, *** *P* < 0.001, compared to Control group. # *P* < 0.05, ## *P* < 0.01, compared to pcDNA3.1-LINC00470 group. GBM-exo, exosomes derived from patients with glioma; MDC, monodansylcadaverin staining; FCM, Flow cytometry

The number of acidic autophagosomes was remarkably less both in the GBM-exo group and pcDNA3.1-LINC00470 group than those in the Control group, according to the results of MDC staining (Fig. 5a, P < 0.05). Western blotting revealed that LC3-I and Beclin1 expression levels were declined, while p62 expression was increased in the GBM-exo group and pcDNA3.1-LINC00470 group when compared to the Control group (Fig. 5b, P < 0.05). Proliferation of glioma cells was strengthened (Fig. 5c–d, P < 0.05) and the number of cells in G1 phase was decreased (Fig. 5e, P < 0.05) in the GBM-exo group and pcDNA3.1-LINC00470 group compared to the Control group. These results further identified that both GBM-exo and LINC00470 overexpression could inhibit the autophagy of U251 and SWO-38 cells but encourage their proliferation.

Moreover, activation of autophagy through Rapa decreased the proliferation of U251 and SWO-38 cells, evidenced by increased acidic autophagosomes (Fig. 5a, P < 0.05), upregulated expression levels of LC3-II/LC3-I and Beclin1, decreased expression of p62 (Fig. 5b, P < 0.05), suppressed proliferative abilities of U251 and SWO-38 cells (Fig. 5c–d, P < 0.05) and increased number of G1-phase cells (Fig. 5e, P < 0.05) in the pcDNA3.1-LINC00470 + Rapa group when compared to those in the pcDNA3.1-LINC00470 group. Together, these results supported that LINC00470 implicated in the proliferation of U251 and SWO-38 through regulating their autophagy.

### LINC00470 and WEE1 competitively bind to miR-580-3p

**Fig. 6:**
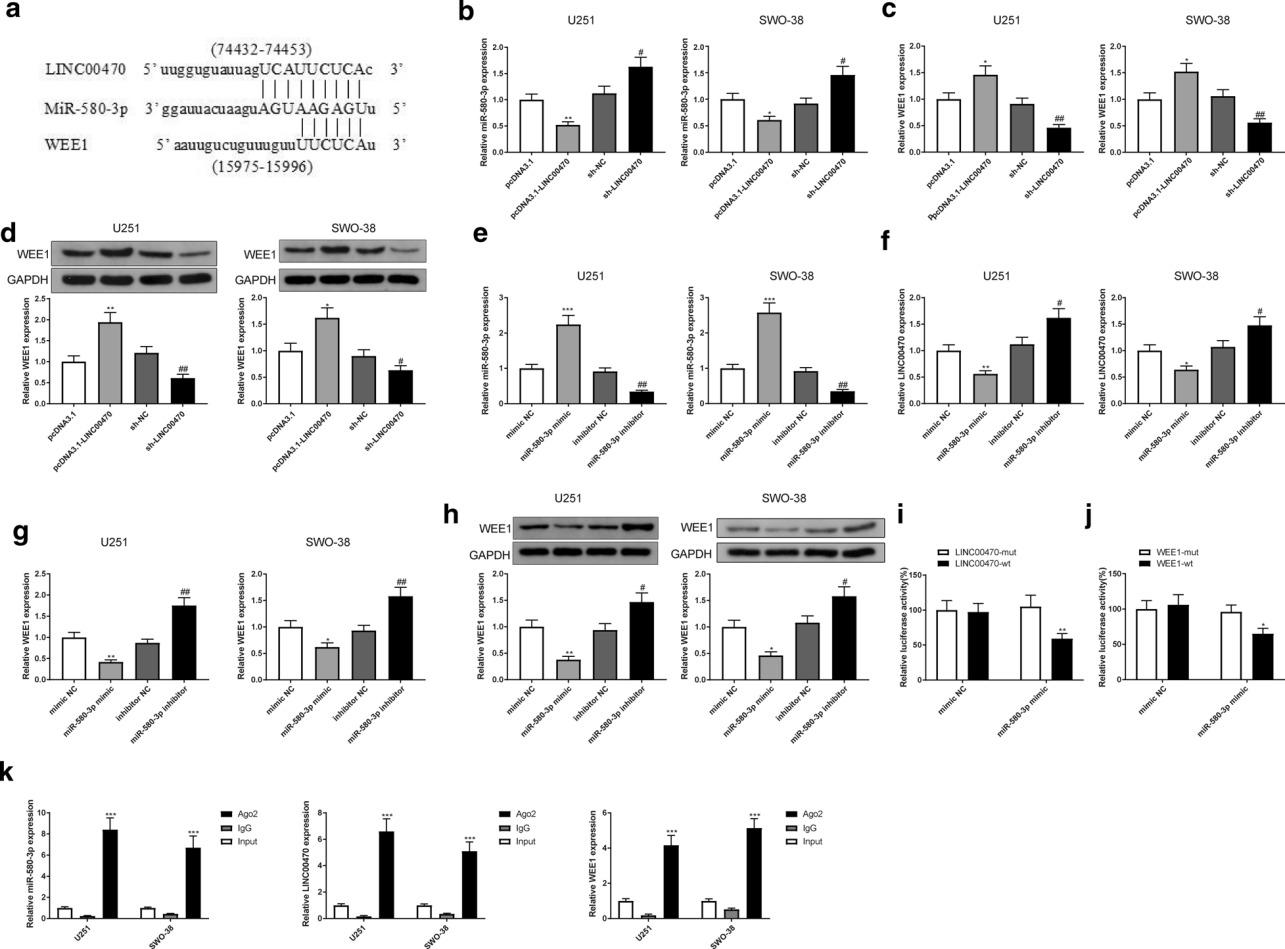
The expression of WEE1 was regulated by LINC00470 via sponging miR-580-3p in glioma cells. Note: The competitive binding sites of LINC00470 and WEE1 with miR-580-3p (**a**). After U251 and SWO-38 cells were transfected with pcDNA3.1-LINC00470 or sh-LINC00470 or their negative controls, the expression of miR-580-3p was examined by qRT-PCR (**b**). The expression of WEE1 was evaluated by qRT-PCR (**c**) and Western blotting (**d**), * *P* < 0.05, ** *P* < 0.01, compared to pcDNA3.1 group, # *P* < 0.05, ## *P* < 0.01, compared to sh-NC group. After U251 and SWO-38 cells were transfected with miR-580-3p mimic or miR-580-3p inhibitor or their negative controls, qRT-PCR evaluated the expression of miR-580-3p (**e**), LINC00470 (**f**) and WEE1 (**g**). Western blotting detected the expression of WEE1 (**h**), * *P* < 0.05, ** *P* < 0.01, *** *P* < 0.001, compared to mimic NC group, # *P* < 0.05, ## *P* < 0.01, compared to inhibitor NC group. Dual luciferase reporter assays were performed to assess the luciferase activity of U251 and SWO-38 cells (**i**–**j**), * *P* < 0.05, ** *P* < 0.01, compared to LINC00470-mut or WEE1-mut group. The binding of miR-580-3p with LINC00470 and WEE1 was detected by RIP (**k**), *** *P* < 0.001, compared to IgG group. NC, negative control; RIP, RNA immunoprecipitation

It has been well-documented that overexpression of WEE1 promotes the proliferation and drug resistance of glioma cells [20, 21]. The binding sites of LINC00470 (ENSG00000132204) and miR-580-3p were predicted through LncBase Predicted v.2 (http://carolina.imis.athena-innovation.gr/diana_tools/web/index.php?r=lncbasev2/index-predicted), and the binding sites of miR-580-3p and WEE1 were predicted by starBase (http://starbase.sysu.edu.cn). According the results, the sites that LINC00470 and WEE1 competitively bind to miR-580-3p are shown in Fig. 6a.

In U251 and SWO-38 cells, transfection of pcDNA3.1-LINC00470 and miR-580-3p inhibitor was followed by downregulation of miR-580-3p and up-regulation of LINC00470 and WEE1 (Fig. 6b–h, P < 0.05), while reverse expression patterns were found in cells transfected with sh-LINC00470 or miR-580-3p mimic (Fig. 6b–h, P < 0.05). These results suggested that LINC00470 could negatively regulate the expression of miR-580-3p, while miR-580-3p could negatively mediate WEE1 expression.

Furthermore, the dual luciferase reporter assay indicated that there was no difference in the luciferase activity of U251 and SWO-38 cells co-transfected with LINC00470-mut and miR-580-3p mimic (Fig. 6i), but the luciferase activity of the cells co-transfected with LINC00470-wt and miR-580-3p mimic was significantly decreased, compared to the negative control group (Fig. 6i, P < 0.01). Meanwhile, miR-580-3p mimic didn’t affect the luciferase activity of U251 and SWO-38 cells transfected with WEE1-mut (Fig. 6j); however, miR-580-3p mimic notably reduced the luciferase activity of U251 and SWO-38 cells transfected with WEE1-wt (Fig. 6j, P < 0.05).

In addition, to further validate the relationship among LINC00470, miR-580-3p and WEE1, we conducted RIP in U251 and SWO-38 cells. It is known that miRNA binds to its target gene through RNA induced silencing complex (RISC) and one of its component is Ago2. Therefore, Ago2 antibody was used for RIP. Based on the results of RIP assay, the levels of miR-580-3p, LINC00470 and WEE1 were enriched in the Ago2 group when compared to those in the IgG group (Fig. 6k, P < 0.001). Taken together, miR-580-3p could bind with LINC00470 and WEE1. LINC00470 can bind to miR-580-3p to regulate the expression of WEE1.

### MiR-580-3p targets WEE1 to affect the proliferation and autophagy of glioma cells

**Fig. 7:**
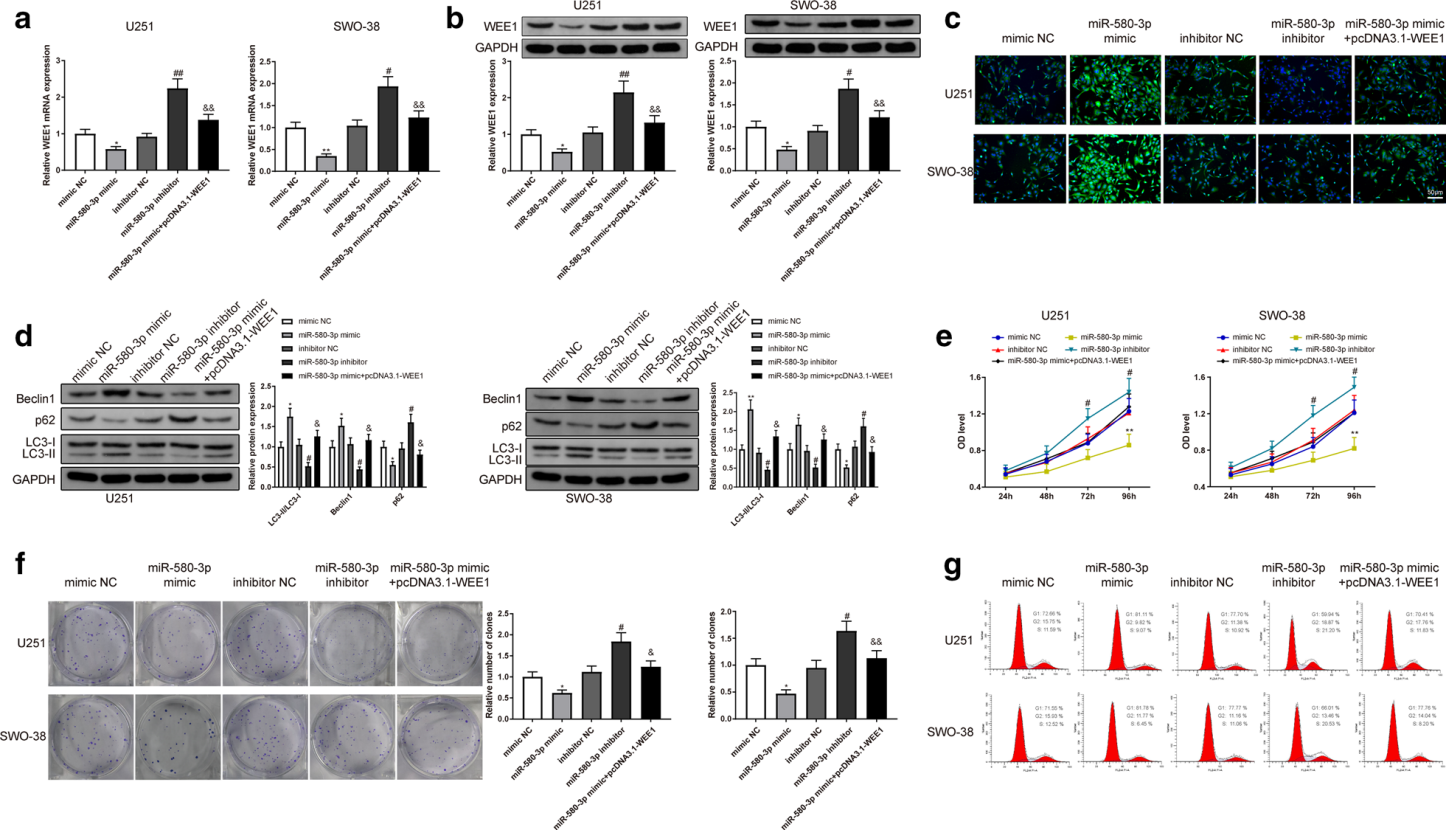
MiR-580-3p targeted WEE1 to promote autophagy and suppress proliferation of glioma cells. Note: After transfected with miR-580-3p mimic or miR-580-3p inhibitor or pcDNA3.1-WEE1 or their negative controls, the expression of WEE1 was analyzed by qRT-PCR (**a**) and Western blotting (**b**). The number of acidic autophagosomes was examined through MDC (**c**) staining. Western blot assay was used to detect the expression of LC3-II/LC3-I, Beclin1 and p62 (**d**). Proliferation of U251 and SWO-38 was evaluated by CCK8 (**e**) and clone formation assay (**f**). FCM assessed cell cycle (**g**). * *P* < 0.05, ** *P* < 0.01, compared to mimic NC group. # *P* < 0.05, ## *P* < 0.01, compared to inhibitor NC group. & *P* < 0.05, && *P* < 0.01, compared to miR-580-3p mimic group. MDC, monodansylcadaverin staining; FCM, flow cytometry

Western blotting and qRT-PCR revealed that WEE1 expression was upregulated in U251 and SWO-38 cells after transfection with pcDNA3.1-WEE1 (Fig. 7a–b, P < 0.05). In the miR-580-3p mimic group, U251 and SWO-38 cells had increased acidic autophagosomes (Fig. 7c, P < 0.05), increased LC3-II/LC3-I and Beclin1 expression, decreased p62 (Fig. 7d, P < 0.05), declined proliferation (Fig. 7e–f, P < 0.05) and less G1-phase cells (Fig. 7g, P < 0.05), compared to the mimic NC group. The reverse results were shown in the miR-580-3p inhibitor group compared to the inhibitor NC (Fig. 7c–g, P < 0.05). Our data demonstrated that cotransfection of miR-580-3p mimic + pcDNA3.1-WEE1 suppressed autophagy and enhanced proliferation of U251 and SWO-38 cells compared to the miR-580-3p mimic group (Fig. 7c–g, P < 0.05). These findings suggested that WEE1 could reverse the inhibitory effects of miR-580-3p on glioma cell progression.

### PI3K/AKT/mTOR pathway on the autophagy of glioma cells

**Fig. 8:**
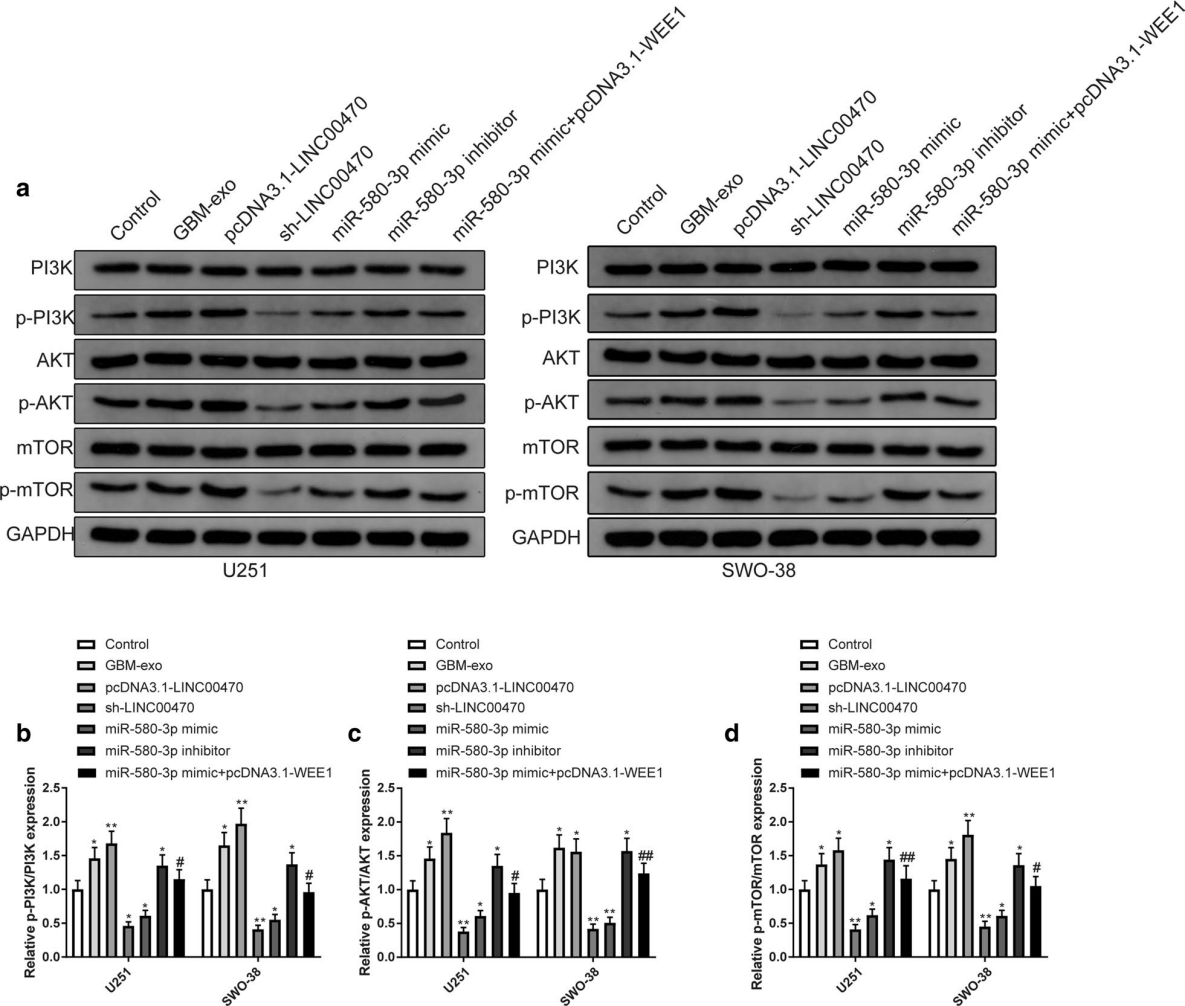
Activation of PI3K/AKT/mTOR pathway inhibited autophagy of glioma cells. Note: miR-580-3p, WEE1 or LINC00470 was overexpressed or inhibited in U251 and SWO-38 cells, and then Western blotting was used to assess the expression levels of PI3K/AKT/mTOR pathway related proteins (**a**) as well as p-PI3K/PI3K (**b**), p-AKT/AKT (**c**), and p-mTOR/mTOR (**d**). * *P* < 0.05, ** *P* < 0.01, compared to Control group. # *P* < 0.05, ## *P* < 0.01, compared to miR-580-3p mimic group; GBM-exo, exosomes derived from patients with glioma

PI3K/AKT/mTOR pathway could regulate cell autophagy whose activation could inhibit autophagy [22]. Thereby, we speculated that LINC00470/miR-580-3p/WEE1 axis may regulate cell proliferation and autophagy through regulating the PI3K/AKT/mTOR pathway. Incubation of GBM-exo or transfection with pcDNA3.1-LINC00470 was followed by increases in the expression levels of p-PI3K/PI3K, p-AKT/AKT and p-mTOR/mTOR in U251 and SWO-38 cells compared to the Control group (Fig. 8a–d, P < 0.05). However, treatment of sh-LINC00470 decreased the expression levels of p-PI3K/PI3K, p-AKT/AKT and p-mTOR/mTOR (Fig. 8a–d, P < 0.05). Transfection of miR-580-3p mimic decreased the expression levels of phosphorylated proteins related to the PI3K/AKT/mTOR pathway and miR-580-3p inhibitor increased the phosphorylated expressions of PI3K/AKT/mTOR pathway related proteins. Co-transfection of miR-580-3p mimic + pcDNA3.1-WEE1 increased the expressions of p-PI3K/PI3K, p-AKT/AKT and p-mTOR/mTOR compared to the miR-580-3p mimic group (Fig. 8a–d, P < 0.05). These results suggested that incubation with GBM-exo or overexpression of LINC00470 or knockdown of miR-580-3p could activate the PI3K/AKT/mTOR pathway.

## Discussion

Glioma is a fatal disease featured by diffusive growth, high invasiveness and infiltration to adjacent brain tissue [16]. Localized treatment for this disease has limited efficiency and therefore glioma demands novel therapeutic approaches [23]. Herein, we identified that exosomal LINC00470 as an oncogene in glioma that promoted the proliferation of glioma cells by inhibiting glioma cell autophagy. More specifically, we identified LINC00470 can bind to miR-580-3p to regulate WEE1 and thereby regulate PI3K/AKT/mTOR pathway, ultimately mediating cell autophagy in glioma.

By detecting the LINC00470 expression in serum of glioma patients, we found that LINC00470 overexpressed in exosomes from serum of glioma patients. Also, the expression of exosomal LINC00470 positively correlated with the clinicopathological characteristics and negatively associated with postoperative survival of glioma patients, supporting its role as a prognosis biomarker for glioma patients. Thereby, we surmised that exosomal LINC00470 participated in glioma progression. To further validate our findings, we established primary glioma model in nude mouse. The results suggested that GBM-exo strengthened tumorigenic ability of U251. To understand its role in autophagy of glioma, we measured the autophagy related proteins in mouse models. Beclin is an initiation protein of autophagosome and LC3 a structural component of the autophagosomes [24], and P62 is an adaptor protein, containing an ubiquition-binding association (UBA) domain and an LC3-interaction region (LIR), that targets ubiquitinated substrates to the autophagosome [25]. LC3 (LC3-I and LC3-II) is essential for the elongation of autophagosomes. LC3-II, localized in both the inner and outer membranes of autophagosomes, increases resulted from LC3-I conversion during autophagy and is viewed as the most reliable marker for quantification of the level of autophagy in cells [26]. In this study, we found that exosomes from serum of glioma patients can inhibit cell autophagy in nude mouse, evidenced by decreased expressions of LC3-II/LC3-I and Beclin1 and increased p62. As exosomes are carriers of multiple factors, it is necessary to ascertain how serum exosomes can regulate cell autophagy in glioma. As expected, sh-LINC00470 increased the number of acidic autophagosomes and the expression levels of LC3-II/LC3-I and Beclin1, while decreased the expression of p62, corroborating that it was LINC00470 that suppressed autophagy and potentiated proliferation in glioma cells.

Autophagy is a controversial program that promotes or inhibits cancer cell death, depending on the cellular context, however, reduced spontaneous autophagy activity is shown in glioma cells, with low expression and high cytoplasmic score of the autophagy-related marker Beclin-1 [27]. Therefore, we hypothesized that exosomal LINC00470 modulated glioma cell proliferation through regulating glioma cell autophagy. Admittedly, Rapa is widely used to activate cell autophagy. After overexpression of LINC00470 and the intervention of Rapa, glioma cell autophagy was strengthened while proliferation was impeded, suggesting that LINC00470 mediated glioma cell proliferation through modulating glioma cell autophagy. Nevertheless, the molecule mechanism of LINC00470 regulating autophagy and proliferation was still unclear.

In the cellular experiments, we found a massive decrease in miR-580-3p expression and a significant increase in WEE1 expression in glioma cells. Through using online bioinformatics prediction and dual luciferase reporter assay, we validated that LINC00470 and WEE1 competitively bound to miR-580-3p and the binding of LINC00470 and miR-580-3p could increase the expression of WEE1. MiR-580 is reported to hinder cell migration in breast cancer [28]. WEE1 activation is considered to be a vital driver of G_2_-M transition through inhibiting phosphorylation of Cdc2, and WEE1 inhibition has been verified to suppress glioma progression in adult nude mouse models via advancing mitosis in cells with damaged DNA [29]. Consistently, in our study, miR-580-3p was reported as a tumor suppressor gene that could impede glioma cell proliferation and augment glioma cell autophagy, while WEE1 could reverse those effects.

In addition, experiments in glioma mouse model unveiled that GBM-exo could activate PI3K/AKT/mTOR signaling pathway, validated by the increased expression levels of p-PI3K/PI3K, p-AKT/AKT and p-mTOR/mTOR. It had been reported that through inactivating PI3K/AKT/mTOR signaling pathway, FK228 sensitizes human glioma cells to TMZ [30]. Upstream signals, including nutrient signaling, growth factors, energy status, oxidative or endoplasmic reticulum (ER) stress and pathogen infection, are integrated by the serine/threonine protein kinase mTOR (mechanistic or mammalian target of Rapa), which acts upstream of the ATG genes, thus controlling autophagy activation [31]. LINC00470 positively regulates AKT activation and suppressed nuclear translocation of phosphorylated AKT [18]. Consistently, GBM-exo, LINC00470 overexpression or miR-580-3p knockdown could activate PI3K/AKT/mTOR signaling pathway, supporting the regulatory role of LINC00470 on PI3K/AKT/mTOR signaling pathway in glioma cells.

## Conclusions

In this study, we demonstrated that LINC00470 secreted by serum exosomes from glioma patients can bind miR-580-3p to regulate WEE1 expression and activate PI3K/AKT/mTOR signaling pathway, thus inhibiting glioma cell autophagy and promoting glioma cell proliferation. Taken together, these findings revealed a novel molecular mechanism of LINC00470 regulating autophagy and proliferation of glioma cells and provided a potential therapeutic target for glioma treatment.

## Data Availability

The datasets used or analyzed during the current study are available from the corresponding author on reasonable request.

## Abbreviations

RIP: RNA immunoprecipitation
TMZ: Temozolomide
EVs: Extracellular vesicles
lncRNA: Long noncoding RNAs
DDR1: Discoidin domain receptor tyrosine kinase 1
HCs: Health checkers
CNS: Central nervous system
TEM: Transmission electron microscope
SPF: Specific pathogen free
H&E: Hematoxylin eosin
IHC: Immunohistochemistry
RIPA: Ratio-immunoprecipitation assay
BCA: Bicinchoninic acid
